# Role of Charge Density of Polycations in DNA Complexation and Condensation

**DOI:** 10.3390/biom15070983

**Published:** 2025-07-10

**Authors:** Jianxiang Huang, Yangwei Jiang, Dong Zhang, Jingyuan Li, Youqing Shen, Ruhong Zhou

**Affiliations:** 1 Institute of Quantitative Biology, College of Life Sciences, Zhejiang University, Hangzhou 310027, China; 2 Department of Physics, Zhejiang University, Hangzhou 310027, China; 3 College of Chemical and Biological Engineering, Zhejiang University, Hangzhou 310027, China; 4 Department of Chemistry, Columbia University, New York, NY 10027, USA

**Keywords:** polycations, hydrophobic interactions, DNA folding, molecular dynamics simulations, electrostatic potential generation, Drew–Dickerson dodecamer, B3LYP

## Abstract

Polycationic gene vectors have been studied extensively for gene delivery, and the charge density of polycations plays a pivotal role in condensing nucleic acids. Recently, we have synthesized two kinds of polycations with varied charge densities: poly(2-(dimethylamino)ethyl methacrylate) (denoted as A100) and a copolymer of 2-(tetramethyleneimino)ethyl methacrylate and 2-(diisopropyl-amino)ethyl methacrylate with a 3:1 feed ratio (denoted as B75D25). Despite its lower charge density, B75D25-based vectors exhibit higher transfection efficiency than A100-based vectors, prompting the hypothesis that hydrophobic interactions, rather than solely high charge density, enhance DNA complexation and gene delivery. This study aims to investigate the molecular mechanisms underlying these differences using molecular dynamics (MD) simulations to study the complexation of DNA with B75D25s and A100s. Our simulations reveal that DNA is quite uniformly covered by B75D25s, and the complexation is not only driven by the electrostatic attraction with DNA but more importantly by the hydrophobic interactions among B75D25s. In contrast, only a small fraction of A100s bind to DNA, which is due to the strong electrostatic repulsion among A100s. Our results reveal the contribution of hydrophobic interactions to the complexation of low-charge-density B75D25s with DNA. These results suggest that high charge density may not be essential for DNA condensation and efficient gene delivery.

## 1. Introduction

Gene therapy has been proven to be a promising therapeutic strategy. It involves delivering therapeutic nucleic acids into target cells to correct genetic disorders [1]. Carriers are generally required to protect nucleic acids from degradation during their blood circulation for in vivo gene therapy [2]. The gene delivery systems (i.e., vectors) broadly fall into two categories: viral vectors and non-viral vectors [3]. Currently, all the gene therapies in clinical use are viral-based [4]. However, viral-vector-based gene therapy has many limitations, such as high cost [5], limited DNA packaging capacity [6], broad tropism [3], immunogenicity [7,8], and carcinogenesis [9]. For instance, the global gene therapy market, reported at USD 2.99 billion in 2021, is anticipated to grow to more than USD 15.68 billion by 2030, achieving a 20.2% compound annual growth rate (CAGR) during the 2022–2030 period [10]. However, the high cost of these therapies, often exceeding USD 1 million per treatment in the US, poses significant affordability challenges for healthcare systems as well as patients [11]. These economic insights highlight the transformative potential of DNA technologies in clinical applications, despite the limitations of viral-vector-based approaches, and underscore the importance of our research in exploring alternative strategies to improve affordability, scalability, and safety. The non-viral gene vector has the potential to overcome the limitations of viral vectors [12,13]. As a representative class of non-viral vectors, polycation-based vectors have been widely explored for efficient gene delivery [14,15,16,17]. Unfortunately, most polycation-based vectors show relatively low transfection efficiency, as compared to the viral vector [3,18]. Thus, it is of great importance to explore possible polycations to fabricate vectors and estimate their transfection efficiencies.

The charge density of polycations is often considered to be an important parameter for polycationic carriers, as it influences the condensation and release of nucleic acids, as well as the enzymatic degradation of nucleic acids, cellular uptake, and cytotoxicity of the delivery vector [3,19,20,21]. In addition to their role in condensing nucleic acids, the charge density of polycations significantly influences the formation of specific DNA shapes, such as toroidal or rod-like structures, which can serve as precursors for advanced functionalization strategies [22,23]. For instance, polycation-induced DNA folding can create compact, well-defined nanostructures that enhance the potential for targeted delivery by facilitating the attachment of targeting ligands or functional moieties. These tailored DNA architectures not only improve the efficiency of gene vectors but also open avenues for precision targeting in therapeutic applications. While sufficient charge density is necessary for polycations to condense nucleic acids, the interaction between nucleic acids and low-charge-density polycations remains underexplored and may offer unique opportunities for modulating DNA shape and functionality. For instance, experimental studies of low-charge-density polycation carriers, e.g., poly glycidyl methacrylate polymers (polyGMA) [20] and poly(N-methyldiethyleneamine sebacate) (PMSC) [21], reported high transfection efficiencies. It has been proposed that the hydrophobic interactions among these polycations are pivotal to the complexation between these polycations with DNA [20,21]. However, detailed information about the interactions between nucleic acids and low-charge-density polycations is still lacking. Hence, it is not clear how other underappreciated factors, e.g., aforementioned hydrophobic interactions among polycations, contribute to the complexation process and the stability of the low-charge-density polycation/nucleic acids complex (polyplex). Recently, we have synthesized two kinds of polycations: poly(2-(dimethylamino)ethyl methacrylate) (denoted as A100) and a copolymer of 2-(tetramethyleneimino)ethyl methacrylate (TMI) and 2-(diisopropyl-amino)ethyl methacrylate (DPA) with a TMI and DPA feed ratio of 3:1 (denoted as B75D25). It is found that B75D25/DNA complexes remain integrated even at low concentrations and retain transfection ability, while A100/DNA complexes easily dissociate and lose transfection ability [24]. Specifically, at N/P = 20, luciferase expression with B75D25 achieved approximately 10^8^ relative light units per milligram (RLU/mg) compared to A100-48K’s 10^7^ RLU/mg, indicating higher transfection efficiency (see Figure 2b from ref [24]). Interestingly, the protonation level of B75D25 and A100 at pH 7 is about 10% and 50% (Figure S1). The charge density of B75D25 is considerably lower than A100’s as well as that of commercially available polyethyleneimine (PEI) [25,26,27]. Based on these observations, we hypothesize that hydrophobic interactions among low-charge-density polycations enhance DNA complexation and polyplex stability, potentially compensating for weaker electrostatic attractions and improving transfection efficiency compared to high-charge-density polycations.

Molecular dynamics (MD) simulations have been conducted to study polycations–nucleic acids complexation and the underlying mechanism [25,26,27,28]. Previous MD studies mainly focused on electrostatically driven complexation and did not consider the possible impact of hydrophobic interactions among low-charge-density polycations. In this work, we use MD simulation to study the complexation of DNA and B75D25s and compare with the complexation of DNA and A100s with higher charge density. We found that B75D25 polycations cover DNA quite uniformly. Moreover, the complexation is not only driven by the electrostatic attraction between DNA and B75D25s but also by the hydrophobic interactions among B75D25s, which is consistent with our previous findings on Z-DNA condensation [29]. On the other hand, only a fraction of A100s are bound to DNA despite the electrostatic attraction between A100s and DNA being relatively stronger. The lower coverage of A100s should be attributed to the strong electrostatic repulsion among them.

## 2. Simulation Systems and Methods

### 2.1. Simulation Systems

**Figure 1:**
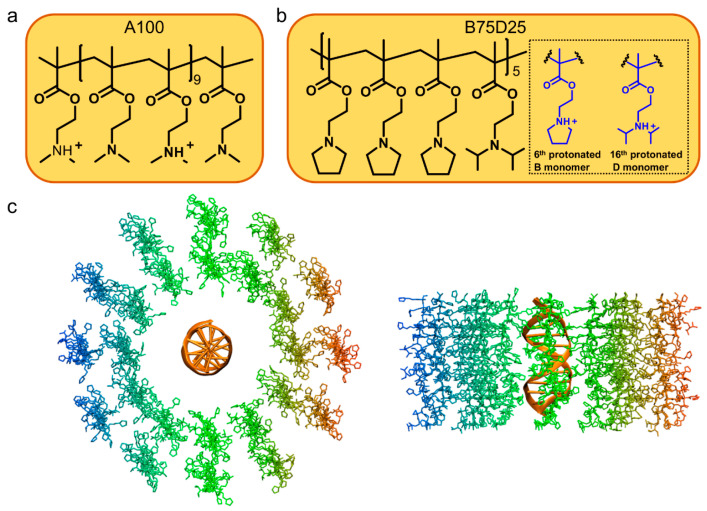
Chemical structures of polycations and the initial configuration of simulation. (**a**) The chemical structure of A100. (**b**) The chemical structure of B75D25. (**c**) The top and side view of the initial setup of B75D25/DNA system. B75D25s are colored by position, using an x-position coloring scheme with a blue–green–red color scale. DNA is shown in brown NewCartoon representation.

The DNA fragment adopted in this study is a Drew–Dickerson dodecamer [30]. It is made of 24 nucleotides (d(CGCGAATTCGCG)_2_). The initial DNA structure was obtained from the Protein Data Bank (PDB entry, 1BNA) [31]. This DNA crystal structure, refined to a residual error of R = 17.8% at 1.9 Å resolution, forms a right-handed B helix with a 19-degree bend over 11 base-pair steps and the central region shows a 36.9-degree rotation per step (9.8 base pairs/turn). The A100 cationic polymer consists of 20 repeated units of 2-(dimethylamino) ethyl methacrylate. Since the protonation ratio of A100 is about 50% at pH 7 (Figure S1), every other monomer of A100 is assigned as protonated (Figure 1a). For simplicity, the TMI and DPA monomers are denoted as B and D. The chemical structure and protonation sites of B75D25 are shown in Figure 1b. As the protonation ratio of B75D25 at pH 7 is about 10% (Figure S1), the 6th and 16th units are assigned as protonated. Additionally, SASA data were generated for both A100 and B75D25, colored by electrostatic potential using the APBS 3.4.1 software [32] (Figure S2). The analysis reveals that A100 exhibits a more positive electrostatic surface than B75D25, consistent with their respective net charges.

The single DNA system is composed of 24 polycations and 1 DNA double strand, therefore the ratio of polycations/DNA is 24:1. It corresponds to the N/P ratio of 20:1 (there are 20 units in each polycation and 12 base pairs in DNA), which is consistent with the experimental condition for DNA and polycation complexation adopted for our previous study [24]. As shown in Figure 1c, there are the inner and outer layers of B75D25s around DNA and each layer contains twelve B75D25s. The center of mass (COM) distances of the inner and outer B75D25 layers from DNA are 3 and 4.5 nm, respectively. A similar initial configuration is built for the A100/DNA system. Two additional systems containing periodically replicated DNA and 24 polycations were constructed. The DNA was aligned along the *Z*-axis, with periodic boundary conditions applied along this axis to approximate an infinitely long DNA molecule in the MD simulation. Moreover, the double DNA system is composed of 48 polycations and two DNA double strands. Specifically, two DNAs were firstly placed in parallel with a COM distance of 4 nm. Subsequently, 48 B75D25s and 48 A100s were inserted randomly into the simulation boxes to obtain the initial simulation configurations (Figure S3).

### 2.2. Simulation Methods and Structural Analysis

Parameters from the general AMBER force field (GAFF) were used to describe the polycations featured in our simulations. The partial charges for polycations were derived by the restrained electrostatic potential (RESP) method [33] to fit the B3LYP/6-31G(d) electrostatic potential generated using the GAUSSIAN 16 software package [34]. Parameters for DNA were taken from the AMBER BSC1 force field [35] and used in combination with the TIP3P explicit water model [36]. Ions (Na^+^ and Cl^−^) were added to neutralize the system and yield an ionic concentration of 150 mM. The detailed information of the systems is summarized in Table S1, including number of DNAs, number of polycations, sizes of the simulated boxes, etc. All molecular dynamic simulations were performed using the GROMACS 2020 [37] simulation package, wherein the temperature (T = 300 K) and pressure (P = 1 atm) were maintained using a stochastic velocity rescaling thermostat [38] and Parrinello–Rahman barostat [39], respectively. Periodic boundary conditions were applied to all the systems in all directions, and use of the LINCS algorithm enabled a standard integration time step of 2 fs [40]. Three independent trajectories of 200 ns in duration were generated for each single DNA system, while MD simulations of the systems with periodic DNA and polycations were performed for 500 ns. In addition, two independent trajectories of 450 ns in duration were performed for each double DNA system. Short-range electrostatic and van der Waals interactions were calculated at a cut-off distance of 1.2 nm, while long-range electrostatic interactions were treated via the particle mesh Ewald (PME) method [41]. All the simulation snapshots were rendered with Visual Molecular Dynamic (VMD) [42].

For the calculation of the potential of mean force (PMF) profile upon pulling one B75D25 chain from the B75D25/DNA complex, the pulling coordinate was defined as the center of mass distance between the COM of the pulled B75D25 chain and the COM of DNA. The selected B75D25 was pulled away from the COM of DNA over 500 ps, using a spring constant of 1000 kJ mol^−1^ nm^−2^ and a pull rate of 0.01 nm ps^−1^. From the pulling trajectory, snapshots were taken to generate the starting configurations for the umbrella sampling [43] windows. A total of 42 windows were selected. In each window, 10 ns of MD was performed for a total simulation of 420 ns adopted for umbrella sampling. Analysis of the PMF calculation was performed with the weighted histogram analysis method (WHAM) [44].

A contact was counted if any two heavy atoms are within 0.4 nm. In addition, the contacted atom pairs were divided into polar–polar, polar–non-polar, and non-polar–non-polar atom pairs. In line with the default classification threshold of an absolute partial charge of 0.25 e for polar and non-polar atoms in docking software Glide, version 6.1 [45], polar atoms in this study are defined as any atom with an absolute value of atom partial charge greater than 0.25 e, while non-polar atoms are defined as any atom with an absolute value of atom partial charge less than or equal to 0.25 e (Figure S4).

## 3. Results and Discussion

### 3.1. Formation of Polycation/DNA Complexes

Initially, the COM distances of the inner and outer B75D25 layers from DNA are 3 and 4.5 nm, respectively. Driven by the attractions to DNA, these polycations moved towards DNA and formed a B75D25/DNA polyplex. Figure S5a shows the final configurations of a B75D25/DNA polyplex. Interestingly, all 24 polycations approach DNA and formed an aggregate surrounding DNA. The thickness of this polycation aggregate can be up to ~2.5 nm. The wrapping of polycations can render excellent protection of DNA: it can reduce the exposure of DNA to the exterior environment. On the other hand, it should be noted that A100 has a similar chemical structure to B75D25 and higher charge density. Therefore, the electrostatic attraction between A100s and DNA should be stronger. However, on average, only about 7 out of 24 A100 polymers bind to DNA while the rest of the A100s are not attached to the formed polyplexes and exist freely in solution (Figure S5b).

**Figure 2:**
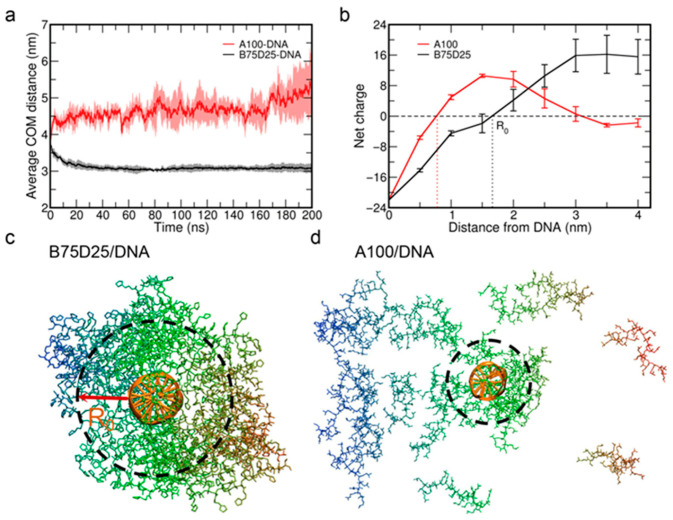
The complexation process of polycations–DNA and the final simulation configurations of the systems. (**a**) Time evolution of the average center of mass (COM) between DNA and the polycations. Shaded error bar represents ± one standard error of the mean. (**b**) Net charge distributions from DNA. Error bar represents standard error of the mean (**c**) Final simulation configuration of B75D25/DNA complex with a dashed circle marking the region within R_0_. (**d**) Final simulation configuration of A100/DNA complex.

The complexation process is further investigated by analyzing the average COM distance between the polycations and DNA. In the case of B75D25/DNA, the average COM distance decreases from ~3.75 nm to ~3 nm at t ≈ 20 ns and remains steadfast (Figure 2a). It illustrates the approaching process of B75D25 to DNA and the formation of a stable polyplex. As for the case of A100/DNA, the average COM distance even slightly increases to ~4.5 nm in the first 5 ns (Figure 2a). The increase in COM distance suggests that some A100s are expelled away from DNA which is largely due to the electrostatic repulsion among A100s, and only a fraction of A100s wrap around DNA (Figure S5b). We also calculated the average COM distance between DNA and A100 chains that are bound to the DNA (Figure S6). The average COM distance decreases from ~3 nm to ~2.2 nm at t = 200 ns. The close COM distance is attributed to the formation of strong electrostatic attractions between DNA and the bound A100 chains.

We then calculate the numbers of polycations in the polyplex, i.e., the polycations involved in wrapping around DNA. As shown in Figure S5a, the wrapping shell of polycations includes both the polymer in direct contact with DNA (i.e., directly contacted polymer) as well as the polymer wrapping on top of these directly contacted polymers. Time evolution of the size of the polycation shell is shown in Figure S7. On average, the size of the polycation shell increases to ~10 for the system containing A100, and only a small fraction of polycations are involved in the wrapping shell. There are ~14 A100 chains that diffuse away from DNA and stay unbound, which is in accordance with the phenomenon of the slightly increased average COM distance between A100s and DNA (Figure 2a). Meanwhile, for the system of B75D25/DNA, the size of the polycation shell rises up to ~24 within 25 ns. In other words, almost all B75D25 polycations are involved in wrapping around DNA.

To evaluate the electrostatic interaction between DNA and polycations, we calculated the net charge distribution surrounding DNA as a function of distance to the DNA surface (Table 1 and Figure 2b). The net charges sum up the charges of the polycation/DNA complex as well as those of ions within this distance. The negative charge on the DNA chain is -22e and, when the net charge reaches zero, we claim the charge on DNA is “neutralized”. The “neutralizing distance” of the B75D25/DNA system was found to be 1.66 nm (R_0_), while the neutralizing distance of the A100/DNA system was 0.78 nm (data were collected over the last 100 ns). The smaller neutralizing distance of the A100/DNA system is consistent with the higher charge density of A100s. As marked in Figure 2c, most of the B75D25 chains are at least partially within the R_0_ distance, while only a small portion of A100 chains are within the R_0_ distance (Figure 2d). The distribution of B75D25 chains partially outside the region indicates that the complexation is not dominated by electrostatic attractions but hydrophobic interactions among B75D25s, which may play a critical role in the stabilization of the B75D25/DNA complex. Notably, we observe interactions between polycations and the DNA termini (Figure S4). To assess the impact of these terminal contacts, we have performed additional simulations using periodic DNA, which restricts polycation binding to the lateral surface of the DNA (i.e., eliminating terminal interactions). Consistent with the findings from Figure S3, B75D25s exhibit greater aggregation toward DNA compared to A100s (Figure S8). For subsequent analyses, we focus on simulations of finite-length DNA, as the inherent rigidity of periodic DNA and the constrained Z-dimension of these systems limit their ability to model biologically relevant conformational flexibility.

Taken together, the wrapping of DNA by A100s is less efficient than that by B75D25s. Since charge density of A100 is higher than that of B75D25, the electrostatic attraction of DNA to A100 should be stronger than to B75D25. In order to better study the underlying mechanism of wrapping ability of B75D25 with relatively weaker electrostatic attraction to DNA, the hydrophobic interactions among B75D25s within the wrapping shell are analyzed in the following subsection.

### 3.2. Hydrophobic Interactions Among B75D25 Polymers and Between B75D25s and DNA

**Figure 3:**
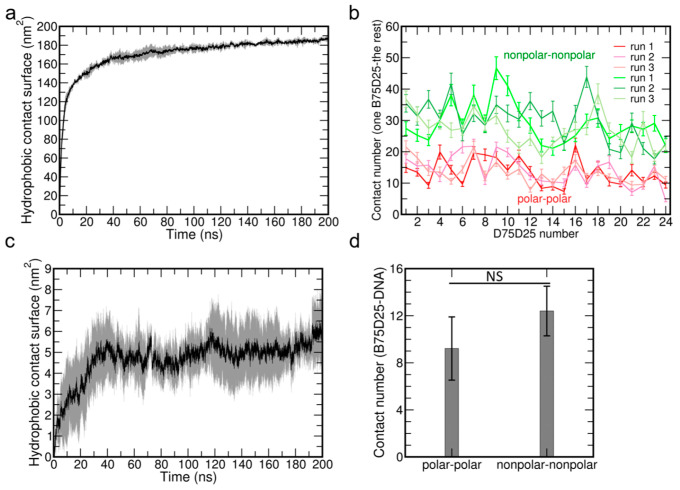
The hydrophobic interactions among B75D25 polymers. (**a**) Time evolution of the hydrophobic contact surface of B75D25s. Shaded error bar represents ± one standard error of the mean. (**b**) Contacted atom pairs among B75D25s (polar–polar atom pairs shown in red lines and non-polar–non-polar atom pairs shown in green lines, cutoff = 0.4 nm). The error bar shows the standard deviation. (**c**) Time evolution of the hydrophobic contact surface between B75D25s and DNA. Shaded error bar represents ± one standard error of the mean. (**d**) Contacted atom pairs between B75D25s and DNA. The error bar shows the standard error. A two-tailed Student’s *t*-test was adopted to determine statistical significance of observed differences in contact number and *p* < 0.05 was considered statistically significant. NS: not significant. Contact numbers from the final 100 ns MD trajectory (5000 frames) were analyzed for statistical significance.

Previous experimental studies about the polycations of polyGMA and PMSC proposed that the hydrophobic interactions among these polycations were pivotal to the complexation between these polycations with DNA [20,21], while the direct evidence about the contribution of hydrophobic interaction among polycations is still lacking. Here, we firstly investigate the hydrophobic interactions among B75D25s by analyzing the hydrophobic contact surface of B75D25s (Figure 3a). The hydrophobic contact surfaces of B75D25s increase sharply from 0 nm^2^ to ~140 nm^2^ in the first 10 ns and then increase gradually to ~180 nm^2^, which reveals the strong hydrophobic interactions among B75D25s.

We further analyze the detailed contacts of each B72D25 with the rest of the B75D25s. More specifically, the average number of contacted atom pairs among B75D25s is calculated. The numbers of contacted polar–polar and non-polar–non-polar atom pairs of each polycation are calculated over the last 100 ns and shown in Figure 3b. It clearly shows that the numbers of contacted non-polar–non-polar atom pairs are significantly higher than the numbers of contacted polar–polar atom pairs. On average, there are about 29 non-polar–non-polar atom pairs between each B75D25 and the other B75D25s, which is considerably higher than that of polar–polar atom pairs (~14). It clearly suggests that hydrophobic interactions dominate the interaction among B75D25s and should facilitate their association. A similar conclusion was previously found in Z-DNA condensation, where hydrophobic contacts of deoxyribose groups result in the stable attraction between two Z-DNA molecules. We also calculate the number of contacting B75D25 chains of each B75D25 chain. On average, each B75D25 contacts with up to ~6 B75D25s in a B75D25/DNA polyplex (Figure S9). Hence, the wrapping B75D25 aggregate can be stabilized by this cooperative hydrophobic interaction of these B75D25 chains.

The potential hydrophobic interactions between B75D25s and DNA is investigated by analyzing the hydrophobic contact surface between B75D25 chains and DNA (Figure 3c). The hydrophobic contact surfaces between B75D25 chains and DNA increase sharply from 0 nm^2^ to ~5 nm^2^ in the first 40 ns and remain steadfast, which reveals the mild hydrophobic interactions between B75D25 chains and DNA. This result implies the potential contribution of hydrophobic interactions to the complexation of B75D25s and DNA. Moreover, we further calculate the non-polar–non-polar and polar–polar atom pairs between B75D25 chains and DNA (Figure 3d). On average, there are about 12 non-polar–non-polar atom pairs between B75D25 and DNA, which is slightly higher than that of polar–polar atom pairs (~9). Consistently, the higher non-polar–non-polar atom pairs support the critical role of hydrophobic interactions in the complexation of B75D25s and DNA. These results suggest that hydrophobic interactions between B75D25s and DNA can somehow facilitate the complexation of DNA with B75D25s.

**Figure 4:**
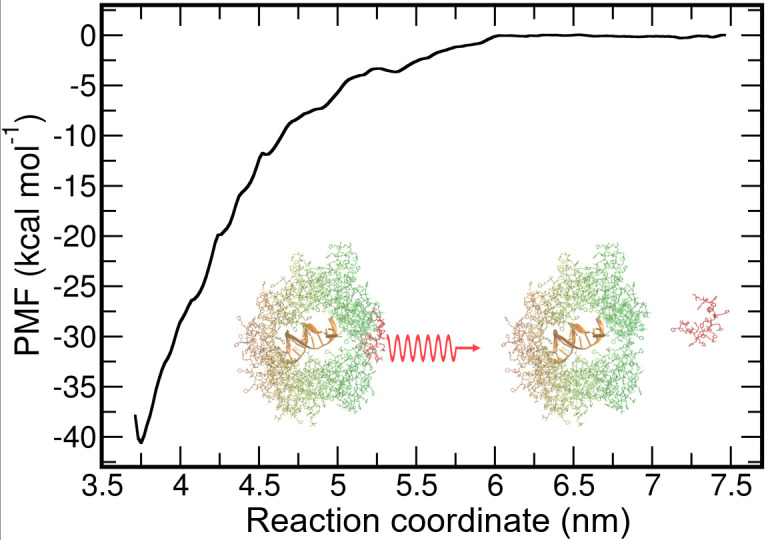
The PMF along the reaction coordinate which is defined as the distance between the COM of the selected B75D25 chain and the COM of DNA. The inset shows the reaction coordinate used for PMF calculations.

In order to further evaluate the hydrophobic interactions, we calculate the potential of mean force (PMF) upon pulling one B75D25 chain away from the rest of the B75D25/DNA complex (Figure 4). The free energy cost of the B75D25 chain disassociation is estimated to be 40.6 kcal mol^−1^ by using umbrella sampling [43] with WHAM [44] (more details are provided in the Simulation Systems and Methods section). A large free energy barrier of 40.6 kcal mol^−1^ supports the strong hydrophobic interactions among B75D25s. Consistently, aggregation of B75D25s is observed in the simulations consisting of only 24 B75D25s (Figure S10).

### 3.3. The Electrostatic Interactions Between B75D25s and DNA

**Figure 5:**
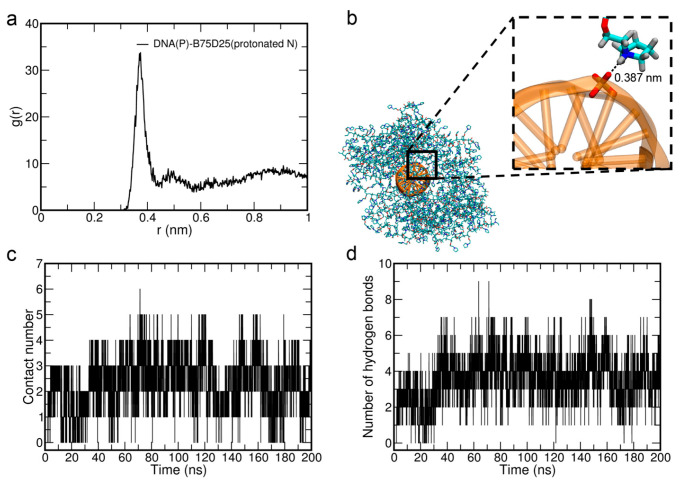
The interactions of DNA and B75D25 polymer. (**a**) The radial distribution function of the protonated amine nitrogen atoms (denoted as protonated N) of the B75D25 polymer around the phosphate phosphorus atoms (denoted as P) of DNA. (**b**) The representative snapshot of DNA(P) interacting with B75D25(protonated N). The colors of P, O, C, H atoms are brown, red, cyan, and grey, respectively. The dashed line marks the distance between DNA(P) and B75D25(protonated N). (**c**) Time evolution of the contact number between DNA(P) and B75D25(protonated N). (**d**) Time evolution of the number of hydrogen bonds between DNA phosphate oxygen atoms and the protonated hydrogen atoms of B75D25 polymers.

We notice that there should be an electrostatic attraction between B75D25s and DNA, which may also help the B75D25s–DNA complexation. We thus analyze the interaction between the protonated amine groups of B75D25s and the phosphate groups of DNA (denoted as N-P interaction). More specifically, the radial distribution function (RDF) of the protonated amine nitrogen atoms of B75D25 polymers around the phosphate phosphorus of DNA is calculated (Figure 5a). The dominant peak at r = 0.38 nm suggests a strong electrostatic attraction between the amine groups of B75D25s and phosphate groups of DNA, and the radius of the first coordination shell is 0.4 nm. In the representative snapshot shown in Figure 5b, the protonated amine group of B75D25 is in close contact with the phosphate group of DNA. These results highlight an essential role of N-P interactions in the stability of B75D25/DNA polyplexes. This phenomenon is similar to previous observations of electrostatic attractions between the cationic residues of the polymers and phosphate groups of DNA [25,26,28,46,47,48].

The number of contacts between the protonated amine groups of B75D25s and the phosphate groups of DNA (i.e., N-P contacts) is also calculated. The number of N-P contacts is calculated by counting the number of appropriate atoms within the radius of the first coordination shell (0.4 nm). The average N-P contact number of the B75D25/DNA polyplex is only 2.5 (Figure 5c). Furthermore, the number of hydrogen bonds between B75D25s and DNA is calculated (Figure 5d). The average number of hydrogen bonds is only 3.7 in the B75D25/DNA polyplex. Furthermore, we performed the simulation of DNA and B75D25 aggregate. The mild attraction leads to the binding of DNA to the B75D25 aggregate (Figure S11).

Additionally, we also calculated the RDF of protonated amine nitrogen atoms of A100 polymers around the phosphate phosphorus of DNA (Figure S12a) and it shows a similar dominant peak to that in Figure 5a. Meanwhile, Figure S12b shows a representative snapshot of the protonated amine group of A100 in close contact with the phosphate group of DNA. Moreover, the average N-P contact number of the A100/DNA polyplex is 5.3 (Figure S12c), which is higher than that of the B75D25/DNA polyplex. The average number of hydrogen bonds between A100s and DNA is 10.4 (Figure S12d), which is higher than that of the B75D25/DNA polyplex. The contact number and hydrogen bond analysis suggest the electrostatic interactions between DNA and polycations are stronger in the A100/DNA polyplex than in the B75D25/DNA polyplex.

Therefore, it is worth noting that the electrostatic attraction between DNA and B75D25 polycations is mild. The mild electrostatic attraction between DNA and B75D25s facilitates their accumulation around DNA, leading to the formation of B75D25 aggregates. This suggests that, despite the moderate attraction, hydrophobic interactions among B75D25s play a crucial role in stabilizing the complexation process. We can further infer from such mild electrostatic attraction between DNA and B75D25 aggregates that the release of DNA from the vector should be efficient in the target cells [19].

### 3.4. Simulations of Double DNA with Randomly Inserted Polycations

**Figure 6:**
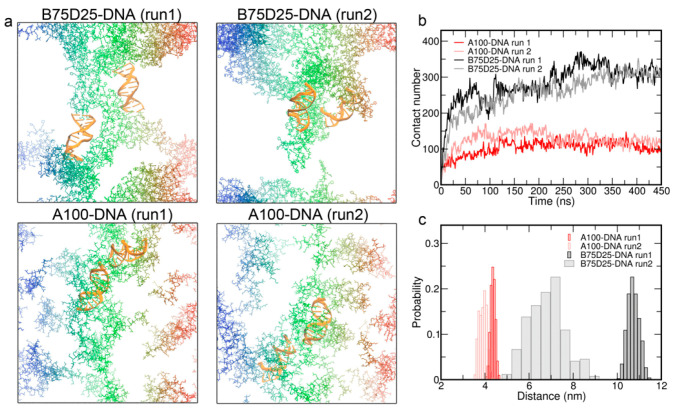
Complexations of double DNA and 48 polycations. (**a**) Final simulation configurations of each double DNA system, obtained from two independent simulations. (**b**) Time evolution of the contact number of DNA and polycations. (**c**) Distribution of COM distance between two DNAs.

To investigate how two DNAs are brought together with the help of polycations, we have performed complexations of double DNA with 48 randomly distributed polycations (Figure S3). The final simulation configurations of two independent simulations for each double DNA system are shown in Figure 6a. It can be observed that DNAs are more closely surrounded by A100 chains than B75D25 chains. Consistent with this observation, the contact number between DNA and A100 chains is ~300, which is much larger than the contact number of ~100 between DNA and B75D25 chains. This result is in line with the stronger electrostatic attractions between A100 and DNA in comparison to those between B75D25 and DNA due to the greater charge density of A100, contrasting with B75D25’s weaker attraction, as evidenced by radial distribution functions (see Figure 5a and Figure S12a). Furthermore, the distribution of the COM distance between two DNAs was calculated over the last 150 ns. The COM distances between two DNAs are much shorter in A100/DNA systems than those in B75D25/DNA systems. It indicates that two DNAs are brought together more closely with A100 chains than B75D25 chains.

## 4. Conclusions

In this study, we perform molecular dynamics simulations to study the impact of charge density on the complexation between polycations and DNA. Low-charge-density B75D25 polycations tend to approach and complex with DNA. Subsequently, B75D25s form an aggregate, which provides DNA with good coverage. The complexation of B75D25 with DNA is not only driven by the electrostatic attraction to DNA but also by the hydrophobic interactions among these low-charge-density polycations. Such hydrophobic-driven formation of polycation assembly also contributes to the overall stability of polycation/DNA polyplexes. Moreover, our findings suggest at least two factors that contribute to the higher transfection efficiency of B75D25-based vectors than A100-based vectors. One is the more uniform and complete DNA coverage of B75D25/DNA polyplexes compared to A100/DNA, which may reduce the exposure of the nucleic acids and thus be favorable to the completeness of the nucleic acids. The other is the relatively weak electrostatic attraction of B75D25s and DNA, which might facilitate the release of nucleic acids from the vectors in the target cells. On the other hand, only a small fraction of A100s, the polycation with much higher charge density, complex with DNA. Much less coverage of DNA is largely attributed to the strong electrostatic repulsion among the A100s. Overall, our study shows that the complexation ability of a polycation is not proportional to its charge density. Hydrophobic interactions among polycations with relatively low charge density may compensate for the relatively weak electrostatic interactions with DNA and form a stable coating layer surrounding DNA and contribute to achieving efficient gene delivery. While our MD simulations provide valuable insights into these polycation–DNA interactions, they are subject to certain limitations. Timescale constraints restrict the ability to fully capture slow conformational changes, such as those involved in long-term polyplex stability, and potential inaccuracies in force field parameters may affect the precise representation of hydrophobic and electrostatic interactions. These factors should be considered when interpreting the simulation results, particularly for extrapolating to experimental or physiological conditions.

To further advance this research, future studies could employ coarse-grained MD simulations and enhanced sampling techniques, such as replica exchange molecular dynamics or metadynamics, to improve sampling efficiency and explore a wider range of polycation compositions and DNA lengths. These approaches would enable more efficient investigation of complexation dynamics over longer timescales and larger systems. Additionally, experimental validation in cellular and in vivo models will be critical to confirm the role of hydrophobic interactions in enhancing cellular uptake and endosomal escape, key barriers in non-viral gene delivery. Moreover, investigating polyplex behavior under physiological conditions, such as in the presence of serum proteins, could further elucidate stability and performance in vivo.

## Figures and Tables

**Table 1:** Net charge distribution from DNA.

Distance from DNA (nm)	A100/DNA	B75D25/DNA
0	−22.0 (0)	−22.0 (0)
0.5	−14.2 (0.420)	−5.63 (0.450)
1.0	−4.50 (0.648)	4.97 (0.634)
1.5	−1.90 (2.45)	10.6 (0.419)
2.0	4.20 (2.82)	9.67 (2.05)
2.5	10.5 (2.95)	4.63 (2.51)
3.0	15.9 (4.26)	0.567 (1.92)
3.5	16.2 (4.98)	−2.37 (0.450)
4.0	15.6 (4.53)	−1.77 (1.05)

Numerals in parentheses are the standard error of the mean.

## Data Availability

The original contributions presented in this study are included in the article/Supplementary Material. Further inquiries can be directed to the corresponding author.

## Supplementary Materials

The following supporting information can be downloaded at: https://www.mdpi.com/article/10.3390/biom15070983/s1, Figure S1: The pH titration curves for the A100 and B75D25 polycations as a function of molar fraction of tertiary amino groups. The pH titration was performed using the same method adopted in the previous report [49]. The result of pKa value of A100 is consistent with previous report (pKa: 7.0–7.3) [50]; Figure S2: The electrostatic potential mapped onto the solvent accessible surface area of A100 and B75D25. Regions of negative potential are colored red, those of positive potential are colored blue, neutral regions are shown in white with scale kT/e = ±10 J/C (k: Boltzmann constant [J/K], T:absolute temperature [K], e: charge of the proton [C]); Figure S3: Initial configurations for the complexations of two DNAs with 48 B75D25s (left) and 48 A100s (right), respectively. The two DNA strands were placed in parallel with a COM distance of 4 nm; Figure S4: Polar atoms of each monomer (highlighted with red circles); Figure S5: Final configurations of B75D25/DNA complex (a) and A100/DNA complex (b), obtained from six independent 200 ns trajectories. The numbers denotes the different indepdent simulations; Figure S6: Time evolution of the average COM between DNA and A100 chains that are bound to the DNA. Shaded error bar represents ± one standard error of the mean; Figure S7: Time evolution of the size of the wrapping shell. The wrapping shell of polycation includes both the polymer in direct contact with DNA (i.e. directly-contacted polymer) as well as the polymer wrapping on top of these directly-contacted polymers; Figure S8: Configurations at t = 0 (a) and 500 ns (b) for the complexations of periodic DNA with 24 A100s. Configurations at t = 0 (c) and 500 ns (d) for the complexations of periodic DNA with 24 B75D25s; Figure S9: Number of contacted B75D25s as a function of each B75D25. The contact among B75D25s is defined as non-hydrogen atoms in close contact with each other (cutoff = 0.4 nm); Figure S10: Aggregation of B75D25 chains. (a) Initial simulation configuration of B75D25s. (b) Final simulation configuration at 200 ns; Figure S11: Binding of DNA and B75D25 aggregate. (a) Initial simulation configuration of B75D25/DNA. (b) Final simulation configuration of B75D25/DNA at 200 ns; Figure S12: Interactions of DNA and A100 polymer; Table S1: Information for the eight systems simulated in this work.
