# Supplementary material for: Role of Charge Density of Polycations in DNA Complexation and Condensation
Source: Biomolecules. 2025 Jul 10;15(7):983. doi: 10.3390/biom15070983 (PMC12292543; doi:10.3390/biom15070983)
Supplement: Supplementary file 1 [file biomolecules-15-00983-s001.zip › biomolecules-3697064-supplementary.pdf]

**Supporting Information**

# Role of Charge Density of Polycations in DNA Complexation and Condensation

Jianxiang Huang[1], Yangwei Jiang[1], Dong Zhang[1], Jingyuan Li[1], Youqing Shen[2], Ruhong Zhou[1,3]*

[1]*Institute of Quantitative Biology, College of Life Sciences, and Department of Physics, Zhejiang University, Hangzhou 310027, China*

[2] *College of Chemical and Biological Engineering, Zhejiang University, Hangzhou 310027, China*

[3]*Department of Chemistry, Colombia University, New York, NY10027, USA*

**Correspondence should be addressed to* rhzhou@zju.edu.cn

**Table S1. Information for the eight systems simulated in this work**

| System | Number of DNA | Number of polycations | Box size ($nm^3$) | Number of water molecules | Number of ions |
|---|---|---|---|---|---|
| B75D25-DNA | 1 | 24 | 12.4×12.4×13.0 | 59774 | 180 $Na^+$, 206 $Cl^-$ |
| A100-DNA | 1 | 24 | 14.0×14.0×9.0 | 53002 | 160 $Na^+$, 378 $Cl^-$ |
| B75D25-periodic DNA | 1 | 24 | 16.0×16.0×3.4 | 22753 | 78 $Na^+$, 104 $Cl^-$ |
| A100-periodic DNA | 1 | 24 | 16.0×16.0×3.4 | 23536 | 78 $Na^+$, 296 $Cl^-$ |
| B75D25 only | 0 | 24 | 12.4×12.4×13.0 | 59774 | 180 $Na^+$, 228 $Cl^-$ |
| B75D25 aggregate-DNA | 1 | 24 | 12.4×12.4×13.0 | 59437 | 180 $Na^+$, 206 $Cl^-$ |
| B75D25-DNA (double) | 2 | 48 | 14.0×14.0×14.0 | 78923 | 249 $Na^+$, 301 $Cl^-$ |
| A100-DNA (double) | 2 | 48 | 14.0×14.0×14.0 | 79541 | 249 $Na^+$, 685 $Cl^-$ |

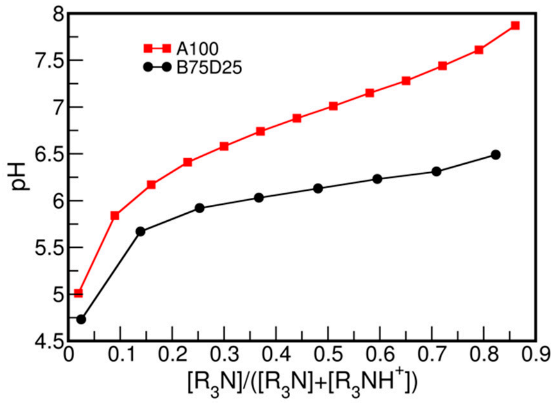


**Figure S1|** The pH titration curves for the A100 and B75D25 polycations as a function of molar fraction of tertiary amino groups. The pH titration was performed using the same method adopted in the previous report[1]. The result of $pK_a$ value of A100 is consistent with previous report ($pK_a$: 7.0–7.3)[2].

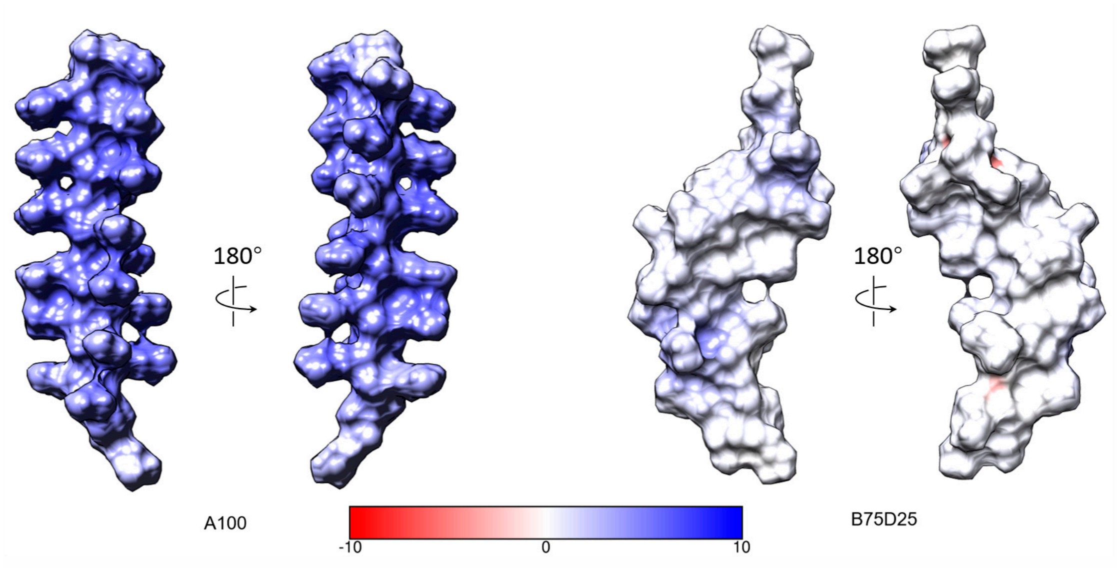


**Figure S2|** The electrostatic potential mapped onto the solvent accessible surface area of A100 and B75D25. Regions of negative potential are colored red, those of positive potential are colored blue, neutral regions are shown in white with scale kT/e = ±10 J/C (k: Boltzmann constant [J/K], T:absolute temperature [K], e: charge of the proton [C]).

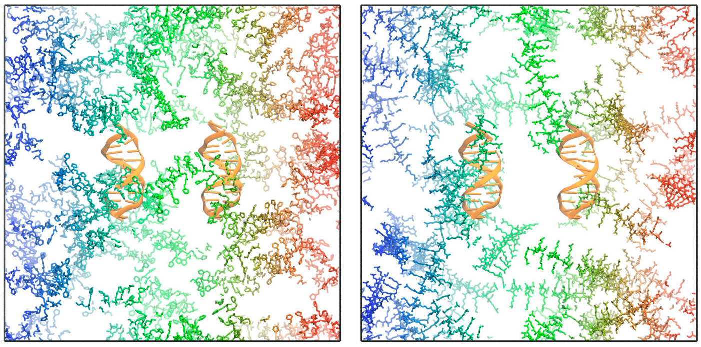

**Figure S3|** Initial configurations for the complexations of two DNAs with 48 B75D25s (left) and 48 A100s (right), respectively. The two DNA strands were placed in parallel with a COM distance of 4 nm.

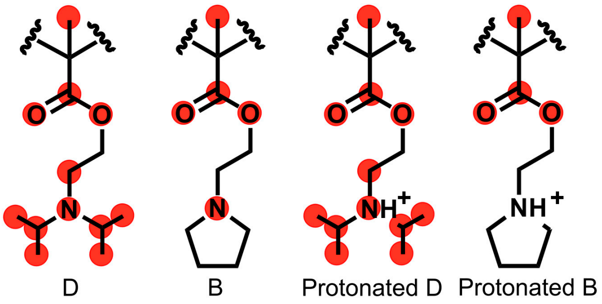


**Figure S4|** Polar atoms of each monomer (highlighted with red circles).

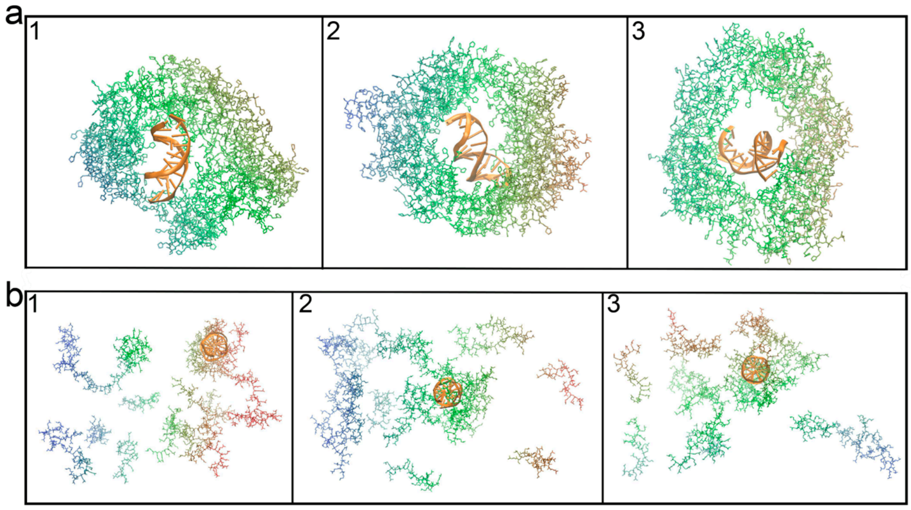


**Figure S5|** Final configurations of B75D25/DNA complex (a) and A100/DNA complex (b), obtained from six independent 200 ns trajectories. The numbers denotes the different indepdent simulations.

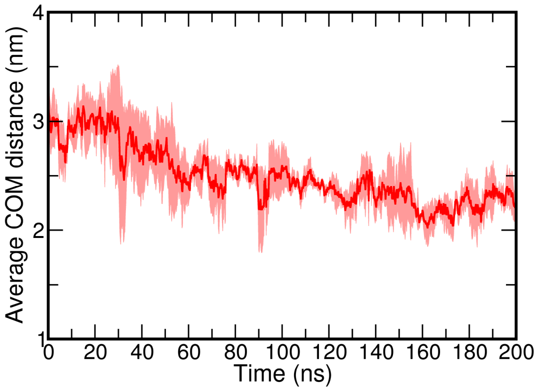


**Figure S6|** Time evolution of the average COM between DNA and A100 chains that are bound to the DNA. Shaded error bar represents ± one standard error of the mean.

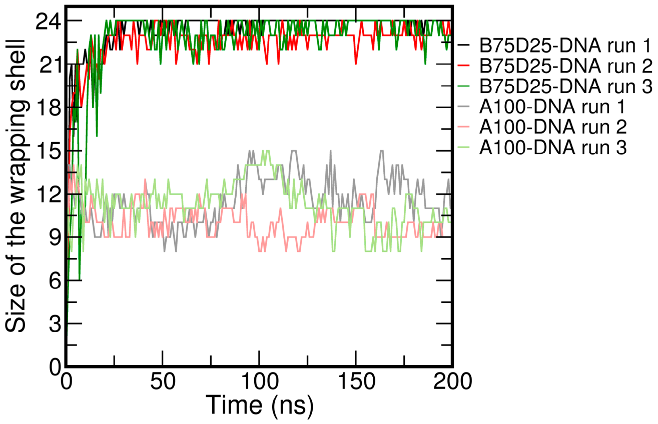


**Figure S7|** Time evolution of the size of the wrapping shell. The wrapping shell of polycation includes both the polymer in direct contact with DNA (i.e. directly-contacted polymer) as well as the polymer wrapping on top of these directly-contacted polymers.

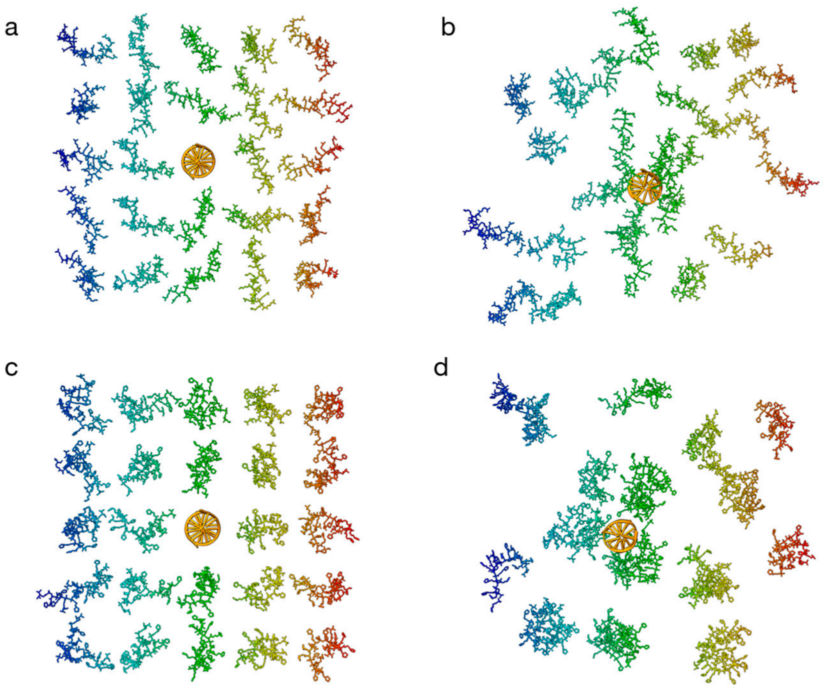


**Figure S8|** Configurations at t=0 (a) and 500 ns (b) for the complexations of periodic DNA with 24 A100s. Configurations at t=0 (c) and 500 ns (d) for the complexations of periodic DNA with 24 B75D25s.

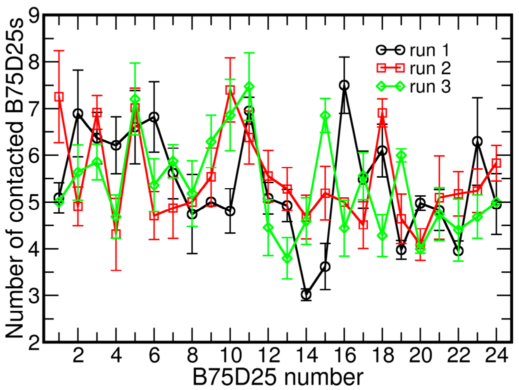


**Figure S9 |** Number of contacted B75D25s as a function of each B75D25. The contact among B75D25s is defined as non-hydrogen atoms in close contact with each other (cutoff = 0.4 nm).

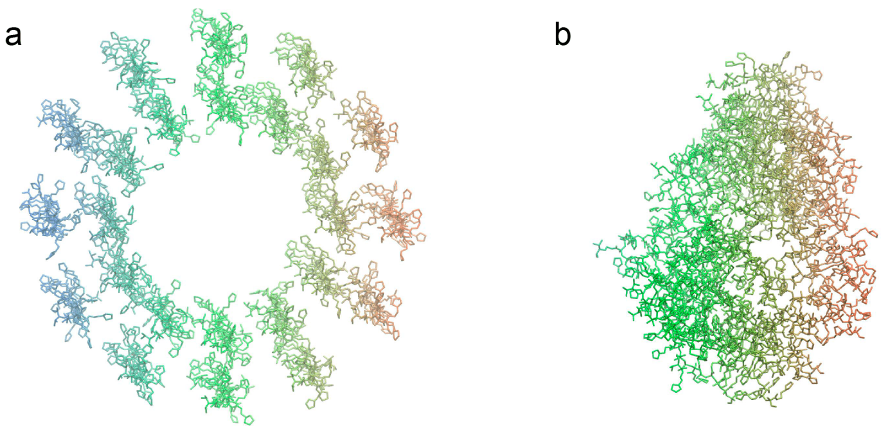


**Figure S10|** Aggregation of B75D25 chains. (a) Initial simulation configuration of B75D25s. (b) Final simulation configuration at 200 ns.

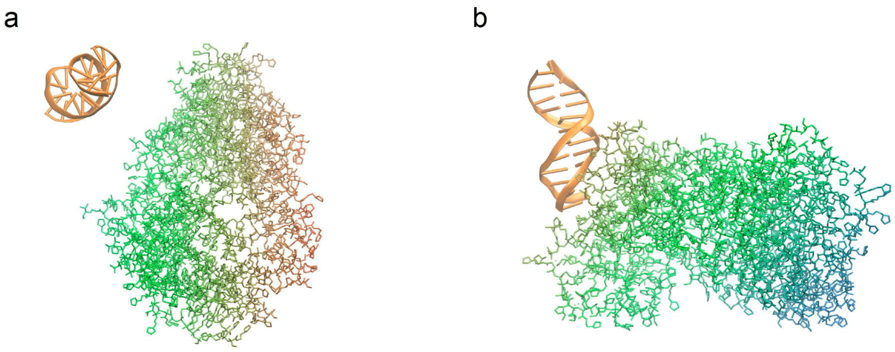


**Figure S11|** Binding of DNA and B75D25 aggregate. (a) Initial simulation configuration of B75D25/DNA. (b) Final simulation configuration of B75D25/DNA at 200 ns.

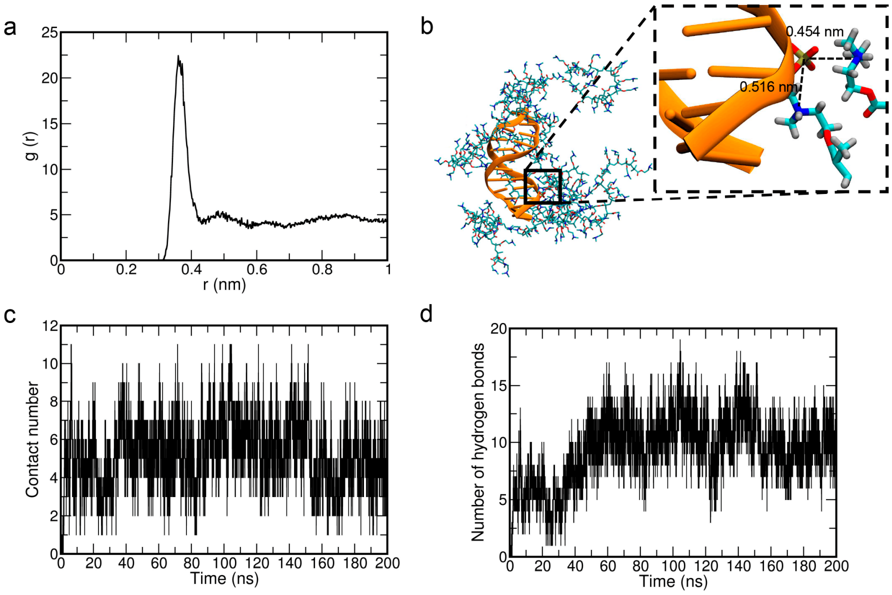


**Figure S12| Interactions of DNA and A100 polymer.** (**a**) Radial distribution function of the protonated amine nitrogen atoms (denoted as protonated N) of the A100 polymer around the phosphate phosphorus atoms (denoted as P) of DNA. (**b**) Representative snapshot of DNA(P) interacting with A100 (protonated N). The colors of P, O, C, H atoms are brown, red, cyan and grey, respectively. (**c**) Time evolution of the contact number between DNA(P) and A100 (protonated N). (**d**) Time evolution of the number of hydrogen bonds between DNA phosphate oxygen atoms and the protonated hydrogen atoms of A100 polymers.

# References

1. Ma, X.; Wang, Y.; Zhao, T.; Li, Y.; Su, L. C.; Wang, Z.; Huang, G.; Sumer, B. D.; Gao, J., Ultra-Ph-Sensitive Nanoprobe Library with Broad Ph Tunability and Fluorescence Emissions. *J Am Chem Soc* **2014**, *136*, 11085-92.

2. Lee, H.; Son, S. H.; Sharma, R.; Won, Y. Y., A Discussion of the Ph-Dependent Protonation Behaviors of Poly(2-(Dimethylamino)Ethyl Methacrylate) (Pdmaema) and Poly(Ethylenimine-Ran-2-Ethyl-2-Oxazoline) (P(Ei-R-Eoz)). *Journal of Physical Chemistry B* **2011**, *115*, 844-860.
